# Transcriptome Analysis Provides Insights into *Potentilla bifurca* Adaptation to High Altitude

**DOI:** 10.3390/life12091337

**Published:** 2022-08-29

**Authors:** Xun Tang, Jinping Li, Likuan Liu, Hui Jing, Wenming Zuo, Yang Zeng

**Affiliations:** 1 College of Life Sciences, Qinghai Normal University, Xining 810008, China; 2 Academy of Plateau Science and Sustainability, Qinghai Normal University, Xining 810008, China; 3 College of Life Science and Technology, Gansu Agricultural University, Lanzhou 730070, China; 4 Qinghai Agricultural Technology Extension Station, Xining 810007, China

**Keywords:** *Potentilla bifurca*, de novo transcriptome sequencing, high altitude, secondary metabolism, flavonoid metabolism

## Abstract

*Potentilla bifurca* is widely distributed in Eurasia, including the Tibetan Plateau. It is a valuable medicinal plant in the Tibetan traditional medicine system, especially for the treatment of diabetes. This study investigated the functional gene profile of *Potentilla bifurca* at different altitudes by RNA-sequencing technology, including de novo assembly of 222,619 unigenes from 405 million clean reads, 57.64% of which were annotated in Nr, GO, KEGG, Pfam, and Swiss-Prot databases. The most significantly differentially expressed top 50 genes in the high-altitude samples were derived from plants that responded to abiotic stress, such as peroxidase, superoxide dismutase protein, and the ubiquitin-conjugating enzyme. Pathway analysis revealed that a large number of DEGs encode key enzymes involved in secondary metabolites, including phenylpropane and flavonoids. In addition, a total of 298 potential genomic SSRs were identified in this study, which provides information on the development of functional molecular markers for genetic diversity assessment. In conclusion, this study provides the first comprehensive assessment of the *Potentilla bifurca* transcriptome. This provides new insights into coping mechanisms for non-model organisms surviving in harsh environments at high altitudes, as well as molecular evidence for the selection of superior medicinal plants.

## 1. Introduction

Plants inhabiting high-altitude environments must face a variety of abiotic stresses, such as low oxygen and carbon dioxide pressure, extreme temperatures, and intense UV radiation [1]. These environmental pressures bring about strong natural selection pressures and drive the evolution of remarkable phenotypes and genetic adaptations, which make the plateau a rich reservoir of species [2,3,4]. The Qinghai-Tibet Plateau is the highest and largest plateau in the world, and is known as the third pole of the earth. It has a variety of environmental types and has become one of the gathering areas of biodiversity, such as snow-capped mountains, salt lakes, and deserts. These environments provide ideal natural laboratories for the study of adaptive evolution [5]. Organisms living on the Qinghai-Tibet Plateau must undergo a series of remarkable genetic evolutionary adaptations to produce a broad range of ecologically adaptive traits. Previous studies on the evolution of genome-level adaptation to the plateau environment have mainly focused on humans and animals, while less research has been done on the mechanism of how plants adapt to the plateau environment at the genome level.

**Figure 1:**
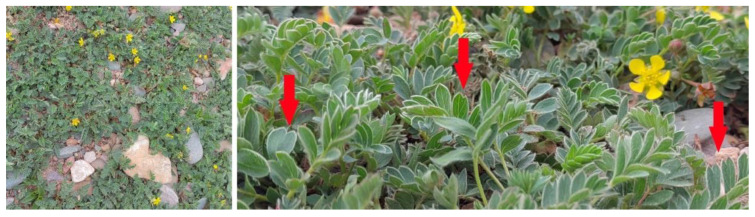
*Potentilla bifurca* plant. The red arrow shows its obovate-elliptic, apex 2-fid leaf type.

*Potentilla bifurca* belongs to the Rosaceae family, and some scholars named it *Sibbaldianthe bifurca* [6,7]. Its typical features are oval or obovate leaves with apex 2-fid or rarely, apex 3-fid (Figure 1). It is widely distributed in Eurasia, including the Qinghai-Tibet Plateau. *P. bifurca* is distributed in various habitats on the Qinghai-Tibet Plateau, including grasslands, wet forests, cold and dry alpine meadows, and gravel slopes above 4000 m, which shows its strong adaptability [8]. It has important medicinal properties, especially in the treatment of diabetes [9]. Species’ genetic structure and genetic diversity information have important guiding significance for formulating effective wild plant resource conservation and management strategies. However, most previous studies have focused on its physiological and morphological characteristics, phylogenetic relationships, and pharmacology [9,10,11]. Although the chloroplast genomes of *P. bifurca* have been resolved, the lack of transcriptome resources has largely hindered the study of adaptive evolution at high altitudes [8,12,13].

In recent years, genome/transcriptome sequencing has proven to be an efficient and rapid method for determining adaptive evolution and differential gene expression in plants. Transcriptome studies have been performed on several Potentilla plants, such as *Potentilla micrantha* [14], *Potentilla anserine* [15], and red-flowered strawberry [16,17]. However, few genome/transcriptome-based studies have been devoted to the study of the molecular mechanisms underlying high-altitude adaptation and evolution in plants [18,19,20]. In this study, we performed RNA-seq to obtain most of the transcript sequences of *P. bifurca*. Positively regulated genes associated with environmental adaptation in *P. bifurca* were identified by genomics comparison with closely related species whose genomes have been sequenced. We aimed to study the mechanism by which *P. bifurca* adapts to the extreme environment of high altitude on the Qinghai-Tibet Plateau at the genomic/transcriptomic level.

## 2. Materials and Methods

### 2.1. Sample Collection

*P. bifurca* was collected in Zeku County (35.234° N, 101.938° E, and 3215masl) and Minhe County (35.835° N, 102.92° E, and 1725masl) of Qinghai Province. To reduce the influence of factors other than the altitude difference between samples, samples were collected at 10:00 a.m. on a sunny day without precipitation for a week, in early August. The collection site was sandy soil on a sunny hillside, and annual plants with a height of 8 ± 2 cm were selected. The *P. bifurca* plants were dug out, carefully removed from the soil, washed thoroughly with distilled water at least three times to clean the adhering soil, blotted dry with sterile filter paper, put into a cryopreservation tube, and immediately stored in liquid nitrogen. The samples collected from Minhe County (1725masl) and Zeku County (3215masl) were control and treatment groups, respectively. The samples were collected in three biological replications. The samples for this study do not involve endangered species.

### 2.2. RNA Extraction and Transcriptome Sequencing

RNA extraction and transcriptome sequencing were performed at Shanghai Applied Protein Technology Co., Ltd. (Shanghai, China). *P. bifurca* whole-plant samples were ground to powder and frozen under liquid nitrogen. The powder, weighing 50 mg, was used for RNA extraction using RNAprep Pure Plant Kit (TIANGEN, Beijing, China). RNA samples were measured with Nanodrop 2000 for sample concentration, and 260/280, 260/230 ratios and Agilent 4150 for RNA integrity. Paired-end libraries were prepared using the ABclonal mRNA-seq Lib Prep Kit (ABclonal, Wuhan, China) according to the manufacturer’s instructions. Then 1 μg of *P. bifurca* total RNA was taken and oligo (dT) magnetic beads were used to adsorb and purify the mRNA. Purified mRNA was fragmented in ABclonal First Strand Synthesis Reaction Buffer. Using the mRNA fragment as a template, random hexamer primers and reverse transcriptase (RNaseH) were used to synthesize the first strand of cDNA, followed by DNA polymerase I and dNTPs to synthesize the second strand of cDNA. The synthesized double-stranded cDNA fragment was ligated to the sequencing primer binding site, index, and P5/P7 for PCR amplification. PCR products were purified and library quality was assessed using an Agilent Bioanalyzer 4150. Finally, sequencing was performed with the Illumina Novaseq 6000 sequencing platform [21].

### 2.3. De Novo Assembly and Functional Annotation

The linker sequences, low-quality reads, and undetermined nucleotide base information ratios greater than 5% were removed from the raw data in FASTAq format based on mass spectral information. Then, clean reads were obtained, which were used for analysis. The Trinity program (http://trinityrnaseq.sourceforge.net/, accessed on 20 August 2021) was used to clean the reads and for de novo assembly [22]. The assembled transcript sequences were compared with NCBI non-redundant protein (Nr), protein family (Pfam), SWISS-PROT protein (SWISS-PROT), KEGG Ortholog database (KEGG), and Gene Ontology database (GO) for the enriched annotation information.

### 2.4. Identification and Annotation of Differentially Expressed Genes 

Each of the unigenes was aligned in the Nr and SWISS-PROT databases, the ORF coding frame information of the aligned transcripts was extracted, and TransDecoder (https://github.com/TransDecoder/, accessed on 21 August 2021) was used to identify the candidate Coding Sequence (CDS) in the transcript sequence. The number of reads per gene was calculated using FeatureCounts software (http://subread.sourceforge.net/, accessed on 21 August 2021), and fragments per kilobase of exon model per million mapped fragments (FPKM) was calculated for each gene, according to the length of the gene. Differential expression analysis of genes between groups was performed using the DESeq2 (http://bioconductor.org/packages/release/bioc/html/DESeq2.html, accessed on 21 August 2021) program, and genes with |log_2_FC| > 1 and Padj < 0.05 were considered to be differentially expressed [23]. The TOP50 DEGs with the most significant up- and down-regulated expression and with clear annotation were screened, and heatmaps were constructed using the Heml v1.0 software (Wuhan, China) [24].

### 2.5. Identification of Transcription Factors and SSRs

All the DEGs were uploaded to the Pfam database (http://pfam.xfam.org/, accessed on 21 August 2021) and PlantTFDB (http://planttfdb.gao-lab.org/index.php, accessed on 21 August 2021) for transcription factor screening and classification based on the extracted annotation information [25]. The DEGs were uploaded to the MISA v2.1 (https://webblast.ipk-gatersleben.de/misa/index.php?action=1, accessed on 10 August 2022) software in batches, and the program parameters were set to repeat at least 10 times for monomers, 6 times for dimers, 5 times for other SSRs, and maximum length of sequence between two SSRs to register as compound SSR was set to 100. The collected SSRs were aggregated and counted [26].

### 2.6. Gene Ontology and KEGG Pathway Analysis

GO and KEGG enrichment analysis of differentially expressed unigenes between groups can elucidate differences between samples at the gene expression level. The Blast2GO program was used to identify differentially expressed unigene ontology (GO) annotations, and the significance level of gene enrichment for each GO term was assessed using Fisher’s exact test [27]. All GO terms were classified using 3 different dimensions, including biological process (BP), molecular function (MF), and cellular component (CC). All unigenes were submitted to KOBAS software for testing the statistical enrichment of differentially expressed genes in the KEGG pathway and assigned to different biological metabolic pathways, including cellular processes environmental information processing, genetic information processing, metabolism, and organic systems [28,29]. The output of KEGG analysis includes KEGG orthology assignments and corresponding Enzyme Commission (EC) numbers along with the metabolic pathways of the unigenes [30].

## 3. Results

### 3.1. Sequencing and Transcriptome Assembly

**Figure 2:**
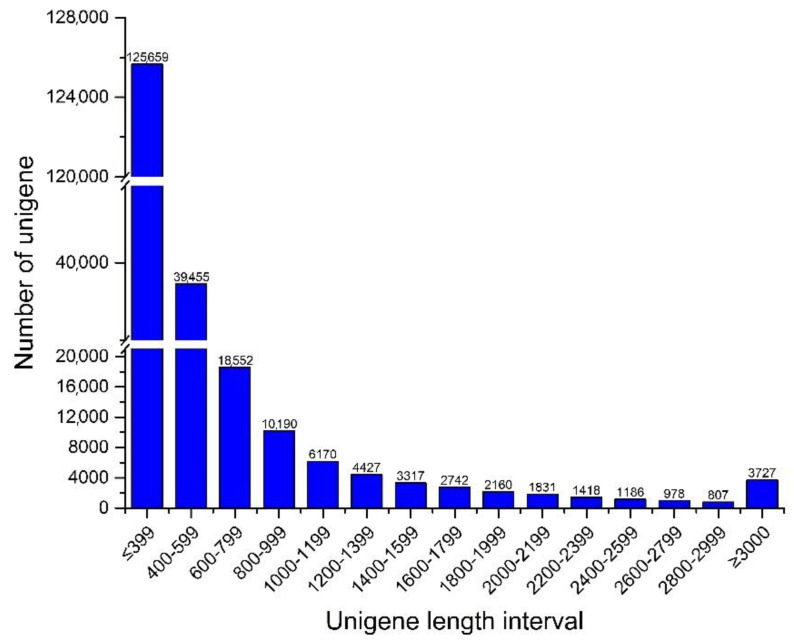
Unigene length distribution in *P. bifurca*.

We detected a total of 405 million clean reads from *P. bifurca* high-altitude and low-altitude samples, accounting for 99.97% of raw reads. These clean reads contained a total of 56.02 GBP clean bases, the average Q20 was 97.51%, and the average GC was 46.45% (Table 1). Clean reads were assembled using Trinity software to obtain a total of 480,582 transcripts. Assembled transcripts showed a rather high N50 value of 1310 bases and an average transcript length of 834 bases. Transcripts were clustered to exclude duplicates, resulting in 222,619 unigenes for subsequent analysis. The average length of unigenes generated by de novo assembly was 595 bp, and the N50 value reached 791 bp. There were 218,906 (98.33%) unigenes with a length of 200 to 3000 bp, which represented the length range of the vast majority of unigenes (Table S1, Figure 2). These findings demonstrate the high quality and rich dataset of sample and transcriptome sequencing.

### 3.2. Sequence Assembly and Annotation

**Figure 3:**
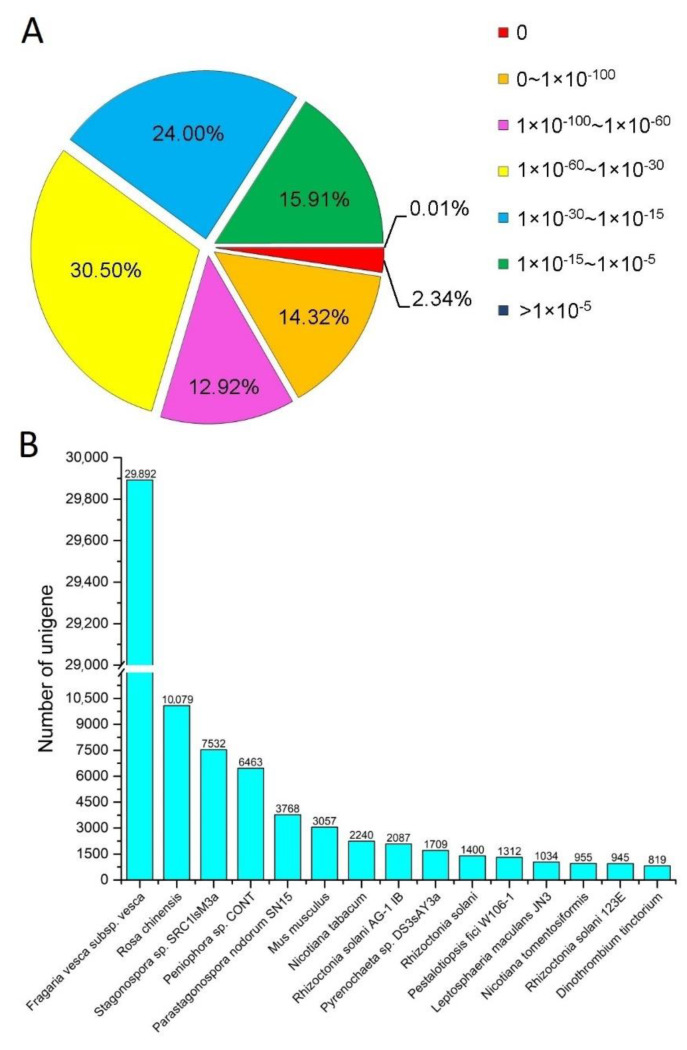
Species classification of the homologous sequences of *P. bifurca* unigenes. (**A**) Frequency distribution of the unigene sequences, according to their E values (cut-off value = 1 × 10^−5^); (**B**) Species distribution of the homologous sequences.

We performed homology searches and functional annotation of all the unigenes in 5 databases, of which 128,327 (57.64%) had homologous genes retrieved in at least one database. Nr and KEGG matched the most homologous genes, 125,599 (56.42%) and 60,488 (27.17%), respectively (Table S2). The alignment of the Nr database showed that 82.7% of the sequence alignments had an E value of less than 1 × 10^15^. Most unigenes shared the significant sequence similarity with *Fragaria vesca* subsp vesca (29,892) and *Rosa chinensis* (10,079), both plants belonging to the same family (Rosaceae) as *P. bifurca* (Figure 3). These gene families have all been reported to be involved in plant responses to abiotic stresses.

### 3.3. Differential Gene Expression Analysis

**Figure 4:**
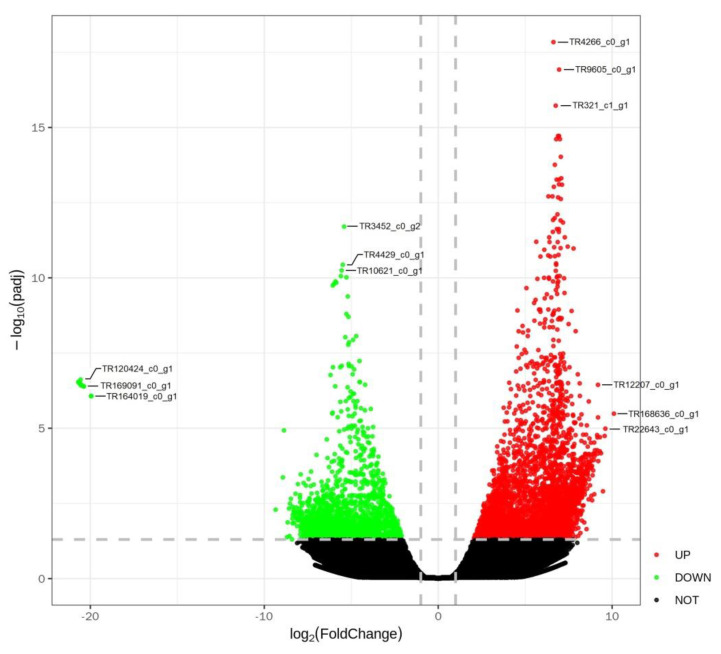
Volcano map of differentially expressed unigenes.

**Figure 5:**
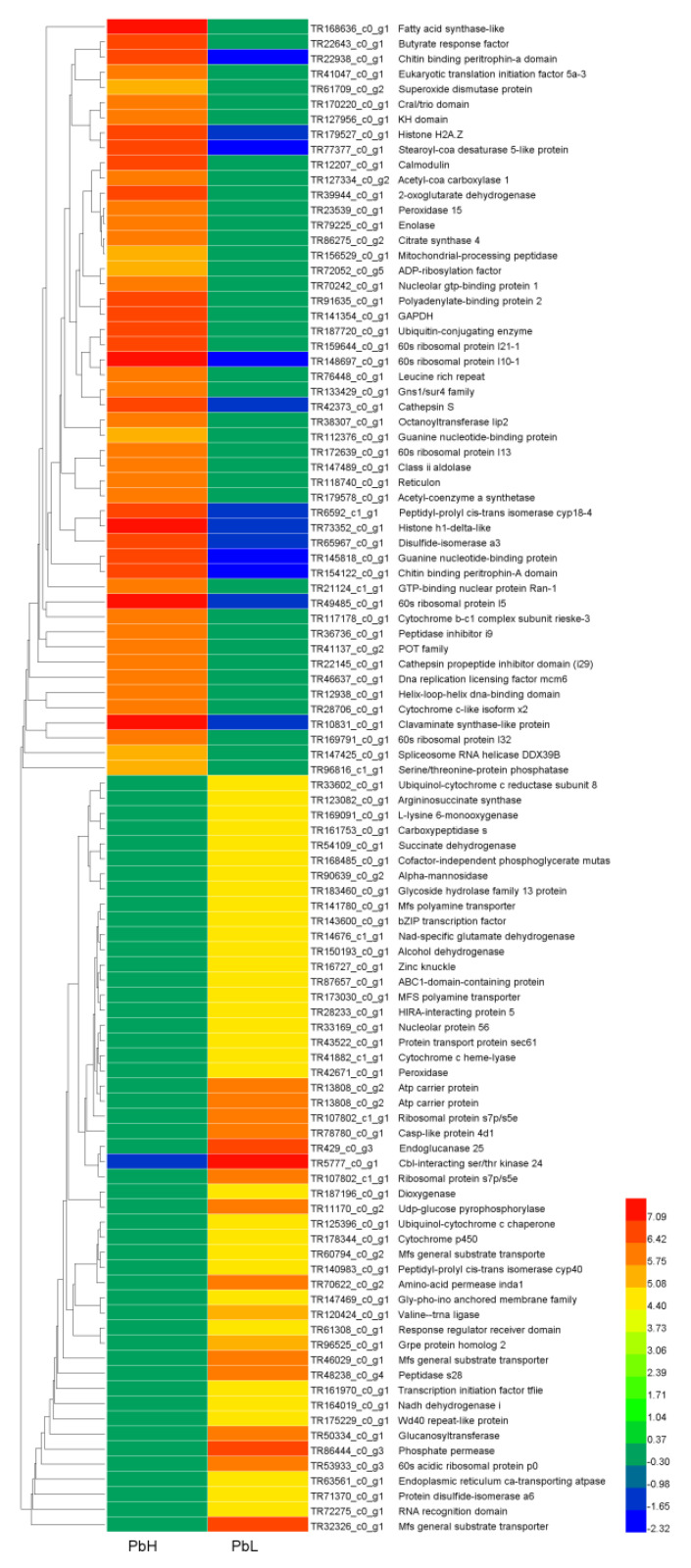
Heatmap showing the top 50 up- and down-regulated genes in high-altitude and low-altitude in *P. bifurca*, with clear annotation.

Differential gene expression analysis showed that 3998 genes were significantly up-regulated (log_2_ fold change ≥ 2 and *p* < 0.05) and 1886 down-regulated genes in high-altitude *P. bifurca* samples relative to low-altitude (log_2_ fold change ≤ −2 and *p* < 0.05) (Figure 4, Table S3). A large number of differentially expressed transcripts indicated the complexity of the mechanism of *P. bifurca* adaptation to a high-altitude environment. We evaluated the expression of unigene by log_2_ fold and selected the top 50 significantly up- and down-regulated genes with clear annotations to construct a heatmap. The results showed that the significantly up-regulated genes in the top 50 included ubiquitin-conjugating enzyme, peroxidase, and superoxide dismutase protein, and these members were often related to plant response to abiotic stresses and scavenging oxygen free radicals [31,32,33]. The top 50 significantly down-regulated genes included succinate dehydrogenase, endoplasmic reticulum Ca-transporting ATPase, cytochrome p450, ubiquinol-cytochrome c reductase, all of which are related to cellular material transport and energy metabolism (Figure 5).

Up-regulated (red) and down-regulated (green) are represented by log_2_ fold change. The black dot represents non-significantly expressed genes.

### 3.4. Functional Classification of DEGs

**Figure 6:**
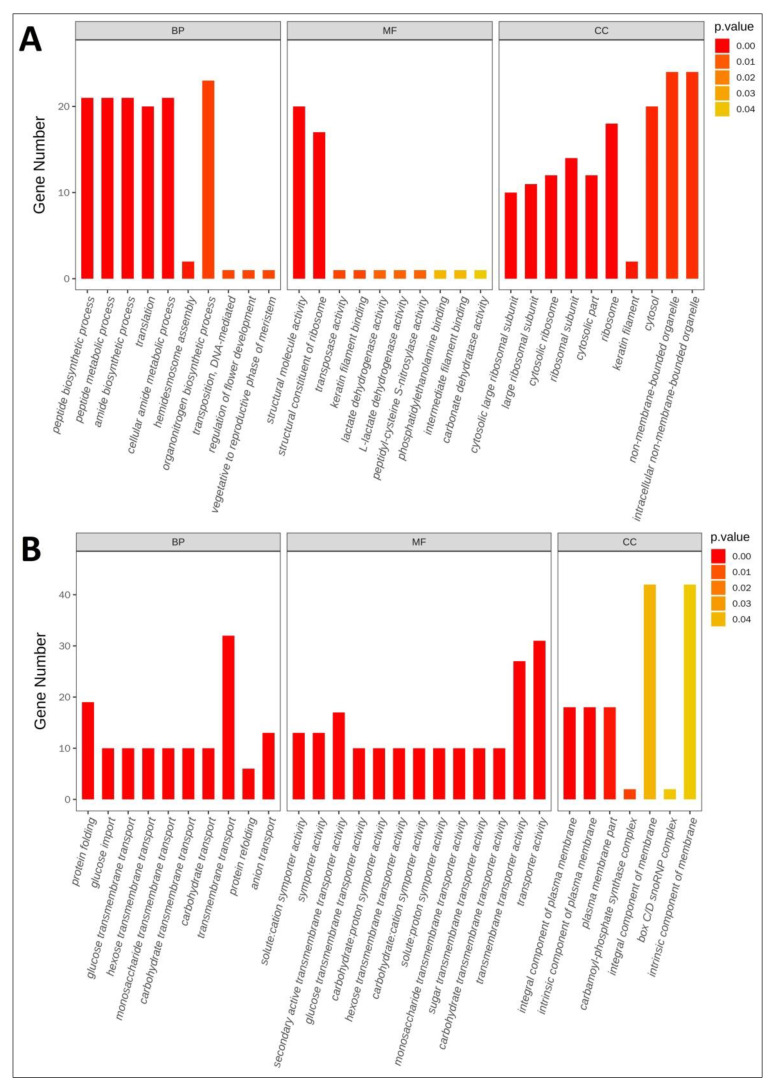
Gene ontology classification of DEGs. (**A**) Up-regulated unigenes; (**B**) Down-regulated unigenes. All unigenes fall into three major functional categories. The Y-axis represents the number of genes in a category; red to yellow represents decreasing *p*-values.

The Gene Ontology (GO) and Kyoto Encyclopedia of Genes and Genomes (KEGG) pathway functional enrichment were performed to identify biological processes or pathways involved in *P. bifurca* adaptation to high altitudes. All 14,183 unigenes were distributed in 64 GO secondary groupings, of which 22, 8, and 14 were involved in biological processes, cellular components, and molecular function categories, respectively (Figure S1). There were 148,844 unigenes mapped to 331 different KEGG pathways (Figure S2). We screened out the GO and KEGG categories with significant differences and these pathways were more indicative of the mechanism of *P. bifurca* adaptation to the high-altitude environment. Among the significantly up-regulated genes, organonitrogen compound biosynthetic process (GO:1901566), non-membrane-bounded organelle (GO:0043228), and intracellular non-membrane-bounded organelle (GO:0043232) groups had the most members, at 23, 24 and 24, respectively. Among the significantly down-regulated genes, transmembrane transport (GO:0055085), integral component of membrane (GO:0016021), and intrinsic component of membrane (GO:0031224) groups had the most members, at 32, 42, and 42, respectively (Figure 6). In the Pfam analysis, the most abundant domain identified was the pentatricopeptide repeat (PPR) family. The top 20 most abundant Pfam domains contained gene families related to abiotic stress response, such as the protein kinase domain, Hsp70 protein, ubiquitin family, etc. (Table 2).

### 3.5. Transcription Factors and SSRs Analysis

**Figure 7:**
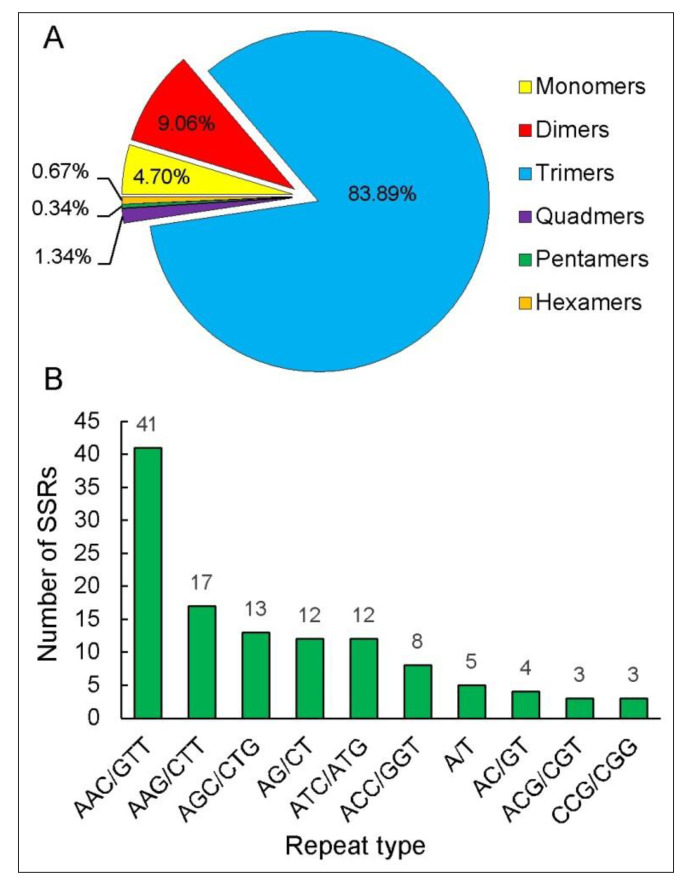
SSR sequences identified in DEGs. (**A**) abundance of different types of SSRs; (**B**) the 10 most abundant SSRs.

Transcription factors play an important role in plant responses to abiotic stresses. We identified 164 transcription factors in differentially expressed unigenes, which were distributed in 27 different families. Among them, 135 (82.32%) transcription factors were up-regulated, including homeobox, zf-C2H2, bZIP, AP2, HLH, and WRKY, and these transcription factors were associated with abiotic stress (Table 3). Simple sequence repeats (SSRs) are among the most important molecular markers in population genetics. This study identified 298 SSRs from 4615 CDSs of differentially expressing unigenes using MISA v2.1 software (Seeland, Germany). The SSR with the largest proportion was trimers (250, 83.89%), followed by dimers (27, 9.06%) (Figure 7A). AAC/GTT repeats were found to be the most frequently occurring SSR in trimers (Figure 7B).

### 3.6. Metabolic PathwayAanalysis by KEGG

**Figure 8:**
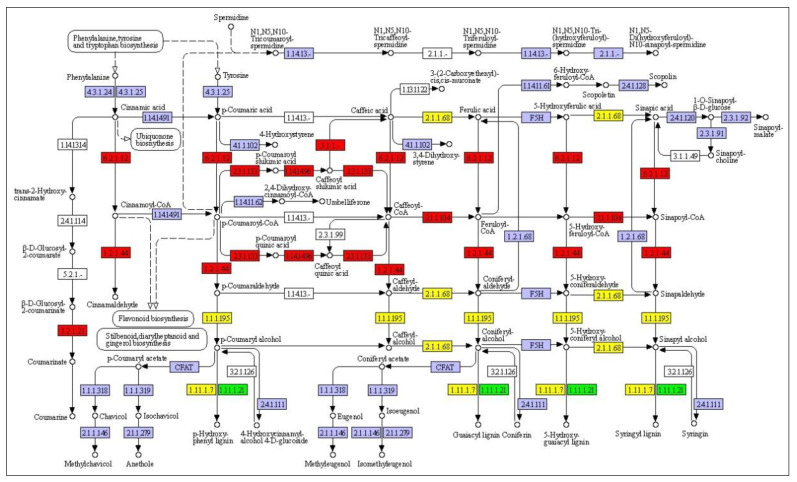
Putative phenylpropanoid pathway in *P. bifurca*.

We enriched DEGs into the KEGG pathway, and the top 20 enriched pathways shared unique molecular pathways, visualized using scatter plots. We found that DEGs up-regulated in *P. bifurca* at high altitudes were mostly enriched in the synthesis of secondary metabolites, such as sesquiterpenoid and triterpenoid biosynthesis (ko00909); cutin, suberine, and wax biosynthesis (ko00073); flavonoid biosynthesis (ko00941); and phenylpropanoid biosynthesis (ko00940) (Figure 8). This shows that the accumulation of these substances was related to the adaptation of *P. bifurca* to the high-altitude environment. Phenylpropane contributed to various aspects of plant responses to abiotic stresses, and it was also the main chemical base of *P. bifurca* as a medicinal plant [9].

Differentially expressed unigenes mapped on the phenylpropanoid biosynthesis pathway. Red represents up-regulated genes, green represents down-regulated genes, and yellow represents both up- and down-regulated genes. The KEGG pathway map k00941 was obtained from http://www.genome.jp/kegg/pathway.html, accessed on 21 August 2021.

**Figure 9:**
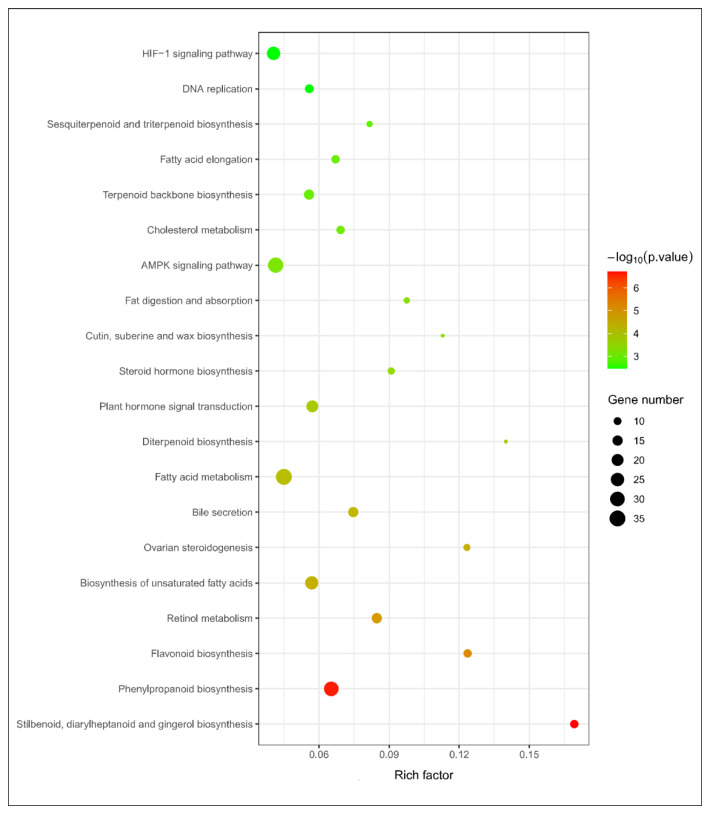
The bubble plot represents the top 20 KEGG pathways of DEGs under positive selection.

Twenty-two members of the phenylpropanoid biosynthesis pathway were significantly up-regulated, accounting for almost one-third of the entire pathway. In contrast, only one member was down-regulated. Interestingly, most of the up-regulated members were upstream of the metabolic pathway, which is crucial to enhancing the pathway (Figure 9).

**Figure 10:**
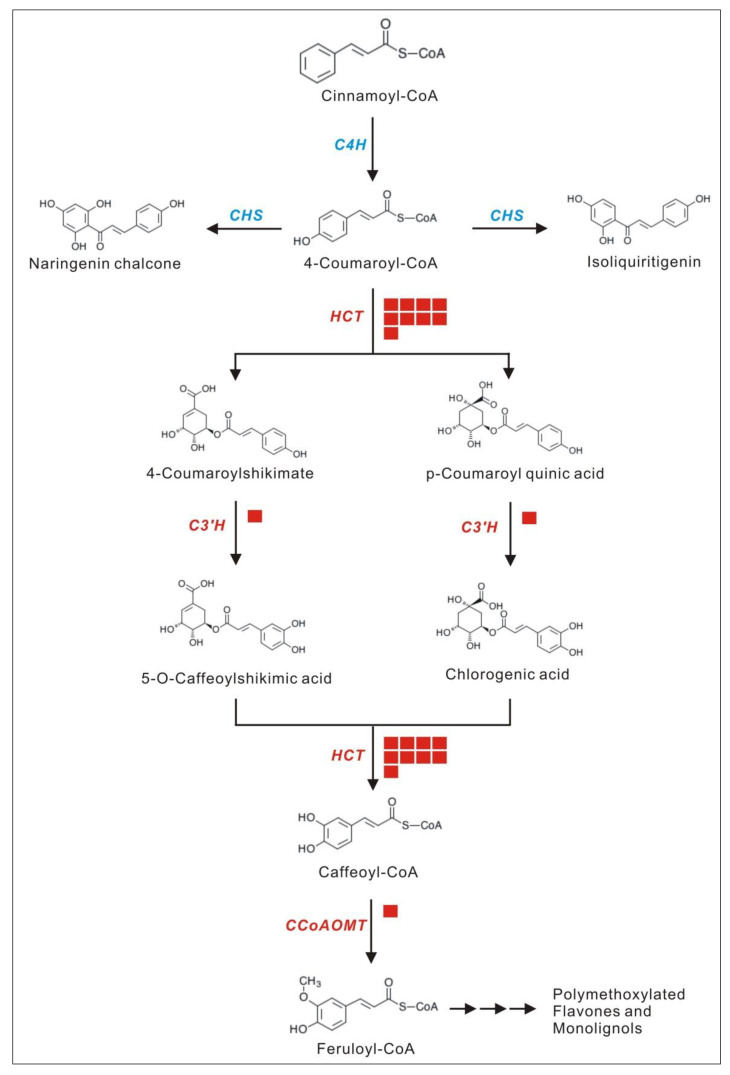
Polymethoxylated flavones and monolignol synthetic pathways. C3’H: 5-O-(4-coumaroyl)-D-quinate 3’-monooxygenase, C4H: cinnamate 4-hydroxylase, CCoAOMT: caffeoyl-CoA O-methyltransferase, CHS: chalcone synthase, HCT: shikimate O-hydroxycinnamoyl transferase.

**Figure 11:**
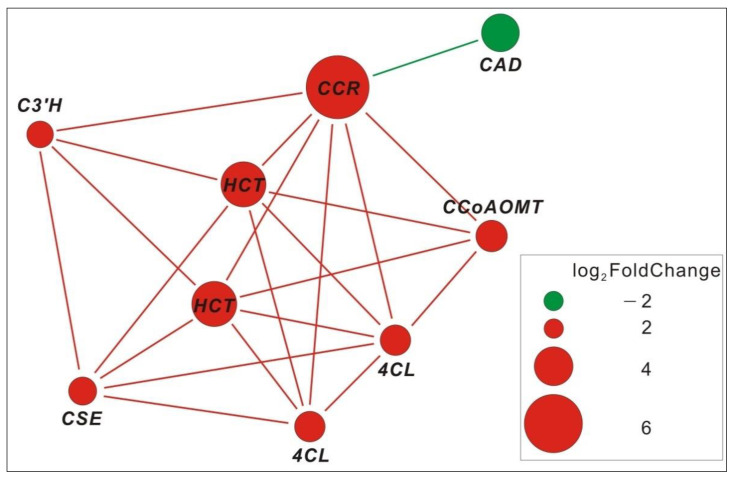
Members interaction in polymethoxylated flavone and monolignol synthetic pathways. 4CL: 4-coumarate-CoA Ligase, C3’H: 5-O-(4-coumaroyl)-D-quinate 3’-monooxygenase, CAD: cinnamyl-alcohol dehydrogenase, CCoAOMT: caffeoyl-CoA O-methyltransferase, CCR: cinnamoyl-CoA reductase, CSE: caffeoylshikimate esterase, HCT: shikimate O-hydroxycinnamoyl transferase.

We also focused on the flavonoid biosynthesis pathway, as it was found that not all flavonoid biosynthesis pathways were up-regulated, but only the polymethoxylated flavones (PMFs) and monolignol synthetic pathways were enhanced, including 5-O-(4-coumaroyl)-D-quinate 3’-monooxygenase (C3’H:), caffeoyl-CoA O-methyltransferase (CCoAOMT), and shikimate O-hydroxycinnamoyltransferase (HCT) (Figure 10). We analyzed the interaction between DEGs, and it was interesting that there was a complex interaction network among the above-mentioned up-regulated genes. This might be the reason behind their up-regulated expression together (Figure 11).

## 4. Discussion

Plants dwelling in high-altitude environments are exposed to a variety of abiotic stresses, including low oxygen, intense UV light, and dramatically changing temperatures, which force these species to undergo a series of adaptive genetic changes, including at the transcriptome level [34,35]. In the present study, for the first time, we assembled the complete de novo transcriptome of *P. bifurca* from both low and high altitudes. Despite the lack of a whole genome sequence, the transcriptome quality of *P. bifurca* produced in this study was still high. Although our study is descriptive, it has added data resources to the genomic study of *P. bifurca*, providing initial insights into the genetic mechanisms of *P. bifurca* adaptation to high altitudes. In addition, the rich genetic data package facilitates our genetic studies of this species.

We detected a total of 405 million clean reads from *P. bifurca*, with a Q20 of 97.51%. After assembly, 480,582 transcripts were obtained, with an N50 value of 1310 bases. We performed a homology search and functional annotation on unigenes, and 57.64% of homologous genes were successfully retrieved. Most of the annotations came from *Fragaria vesca* subsp. vesca, a plant in the same family as *P. bifurca*. This shows the consistency of genetic evolution and morphological classification of *P. bifurca*. These findings demonstrate the high quality of sample and transcriptome sequencing.

There were 3998 genes significantly up-regulated and 1886 down-regulated genes in high-altitude *P. bifurca* samples relative to low-altitude *P. bifurca*. We performed a detailed analysis of these DEGs. We found a large number of abiotic stress-related members from the up-regulated top 50 DEGs, such as peroxidase and superoxide dismutase proteins, which proved to be critical in scavenging ROS generated by abiotic stress, while ubiquitinated members that play a role in clearing damaged proteins were found [36,37]. Plants often choose to turn off growth-related gene expression and turn on stress-related gene expression when they experience abiotic stress. For example, we found members involved in substance transport among genes that were significantly down-regulated, in GO analysis.

Transcription factors play a key role in the regulation of gene expression by binding to cis-acting elements to regulate the transcription efficiency of the target genes. A transcription factor family contains many members, their functions are different, but most of them tend to be the same. We found 164 transcription factors in DEGs, the vast majority of which were up-regulated. Among these up-regulated transcription factors, many transcription factor families are closely related to abiotic stress. For example, WRKY transcription factors, which are a class of transcription factors specific to plants, are named after containing a DNA-binding domain of 60 amino acids. WRKY is involved in drought stress, extreme temperature stress, salt stress, UV stress, etc. [38,39]. We found 14 AP2 members in *P. bifurca*, all of which were up-regulated in response to high-altitude environmental stimuli. AP2 is a large class of transcription factors mainly found in plants, including DRE-binding proteins [40,41]. AP2 has been shown to bind to the dehydration-responsive element/C-repeat cis-acting element and be involved in various types of abiotic stresses, including low temperature [42], drought [43], and hypoxia [44].

Accumulation of secondary metabolites, such as lignans and waxes that reduce water loss and protect against UV rays, benefits plants against abiotic stresses. The phenylpropanoid biosynthetic pathway is activated, leading to the accumulation of various phenolic compounds with the potential to scavenge harmful reactive oxygen species. The polymethoxy flavonoid pathway was enhanced under various stresses in high-altitude environments, possibly due to the role of the phenolic hydroxyl groups of flavonoids in scavenging ROS. Interestingly, we found in previous studies that the main medicinal compounds of *P. bifurca* are flavonoids. The various stress environments in high-altitude areas promote the accumulation of abundant and diverse secondary metabolites by plants to resist stress, which is precisely the chemical basis for plants as natural medicines [45]. This provides a direction for finding the most effective *P. bifurca* botanical source. Proteins are the most critical molecules for achieving biological functions. They often require the action of some small molecules or other proteins to activate. Enzymes in the same metabolic pathway can often interact to enhance the metabolic pathway [46]. We found that multiple members of the polymethoxy flavonoid pathway could interact. This further proves that the polymethoxy flavonoid pathway was enhanced.

Simple sequence repeats are among the most commonly used molecular markers in the study of organism genetic diversity, environmental adaptive genetic structure, and evolutionary laws [47]. SSR markers that can be exploited in *P. bifurca* were not observed. In this study, SSR sites were searched using the transcriptome sequence information of *P. bifurca*, and a total of 298 SSR sites were identified in the CDS of DEGs, of which trinucleotides (83.89%) were most frequently found as repeat motifs in *P. bifurca*. This phenomenon is similar to most plants [48,49]. These SSR motifs may be potential candidates for the development of transcript-based microsatellite markers useful for analyzing molecular mapping, marker-assisted selection, and functional genetic variation in *P. bifurca* [50,51].

## 5. Conclusions

This is the first reported high-quality de novo assembly of the *P. bifurca* transcriptome. Our findings provide preliminary molecular insights and a rich data package on the adaptation of *P. bifurca* to high-altitude environments. A total of 480,582 transcripts and 222,619 unigenes were generated in this study, and approximately 57.64% of the unigenes were annotated and functionally classified. Pathway analysis revealed that a large number of DEGs encode key enzymes involved in secondary metabolites, including phenylpropane and flavonoids. In addition, a total of 298 potential genomic SSRs were identified in this study, the first report of its kind, which provides information on the development of functional molecular markers for genetic diversity assessment. In conclusion, our study will enrich the genomic resources of *P. bifurca*, lay a foundation for further research on the molecular mechanism of *P. bifurca* adaptation to the alpine environment, and provide molecular evidence for the selection of an excellent medicinal plant.

## Figures and Tables

**Table 1:** Summary statistics of the RNA sequencing results.

Group	Replicate	Raw Reads	Clean Reads	Clean Bases (Gbp)	Q20 (%)	Q30 (%)	GC (%)
PbH	1	64,595,390	64,583,098	8.94	97.34	91.91	47.2
	2	70,717,906	70,697,140	9.77	96.82	90.47	47.93
	3	53,381,820	53,368,334	7.35	97.85	93.44	46.1
PbL	1	51,198,370	51,187,944	6.99	98.2	94.42	45.5
	2	62,122,516	62,109,606	8.59	96.99	90.99	46.2
	3	103,556,534	10,3521,292	14.38	97.83	93.34	45.74

PbH: The *P. bifurca* sample collected from high altitude; PbL: The *P. bifurca* sample collected from low altitude.

**Table 2:** Top 20 Pfam domains in differentially expressed unigenes.

Pfam ID	Pfam Description	Number
PF13041, PF01535, PF12854, PF13812	Pentatricopeptide repeat family	2639
PF12799, PF00560, PF13855	Leucine-rich repeats	1710
PF00069	Protein kinase domain	1402
PF07714	Protein tyrosine kinase	1287
PF00071, PF08477	Ras family	763
PF00076	RNA recognition motif	550
PF00083	Sugar (and other) transporter	511
PF07690	Major facilitator superfamily	482
PF00067	Cytochrome P450	476
PF00012	Hsp70 protein	425
PF00400	WD domain	419
PF17177	Pentacotripeptide-repeat region of PRORP	390
PF00106	Short-chain dehydrogenase	372
PF13561	Enoyl-(Acyl carrier protein) reductase	353
PF00931	NB-ARC domain	332
PF00025	ADP-ribosylation factor family	313
PF00005	ABC transporter	305
PF00271	Helicase-conserved C-terminal domain	292
PF00240	Ubiquitin family	286
PF00153	Mitochondrial carrier protein	281

**Table 3:** Type and number of transcription factor families.

Family	Total	Positive	Negative
Homeobox	24	21	3
zf-C2H2	22	14	8
bZIP_2	17	12	5
bZIP_1	15	9	6
AP2	14	14	0
HLH	14	14	0
NAM	9	8	1
B3	8	8	0
SRF-TF	6	5	1
WRKY	6	6	0
HSF_DNA-bind	4	2	2
Ets	3	3	0
CP2	2	0	2
CSD	2	2	0
E2F_TDP	2	2	0
GATA	2	2	0
Pou	2	2	0
SBP	2	2	0
zf-C4	2	2	0
CUT	1	1	0
DDT	1	0	1
Forkhead	1	1	0
Not1	1	1	0
Runt	1	1	0
STAT bind	1	1	0
zf-BED	1	1	0
zf-Dof	1	1	0

## Data Availability

The article contains data sets supporting conclusions and complete protocol descriptions and will be made available to any qualified researcher without reservation. The transcriptome data package of *Potentilla bifurca* is accessible in the Genome Sequence Archive https://ngdc.cncb.ac.cn/gsa, accessed on 14 August 2022) under the accession number CRA007683.

## Supplementary Materials

The following supporting information can be downloaded at: https://www.mdpi.com/article/10.3390/life12091337/s1, Additional supporting information may be found in the online version of this article. Table S1: Statistics of assembled transcripts and unigenes; Table S2: Summary of the annotated and assembled sequences of *P. bifurca*; Table S3: Differentially expressed genes at high altitude relative to low altitude in *P. bifurca*; Figure S1: Gene ontology classification of unigenes; Figure S2: KEGG classification of unigenes.
