# Supplementary figures and images for: Transcriptome Analysis Provides Insights into Potentilla bifurca Adaptation to High Altitude
Source: Life (Basel). 2022 Aug 29;12(9):1337. doi: 10.3390/life12091337 (PMC9503701; doi:10.3390/life12091337)

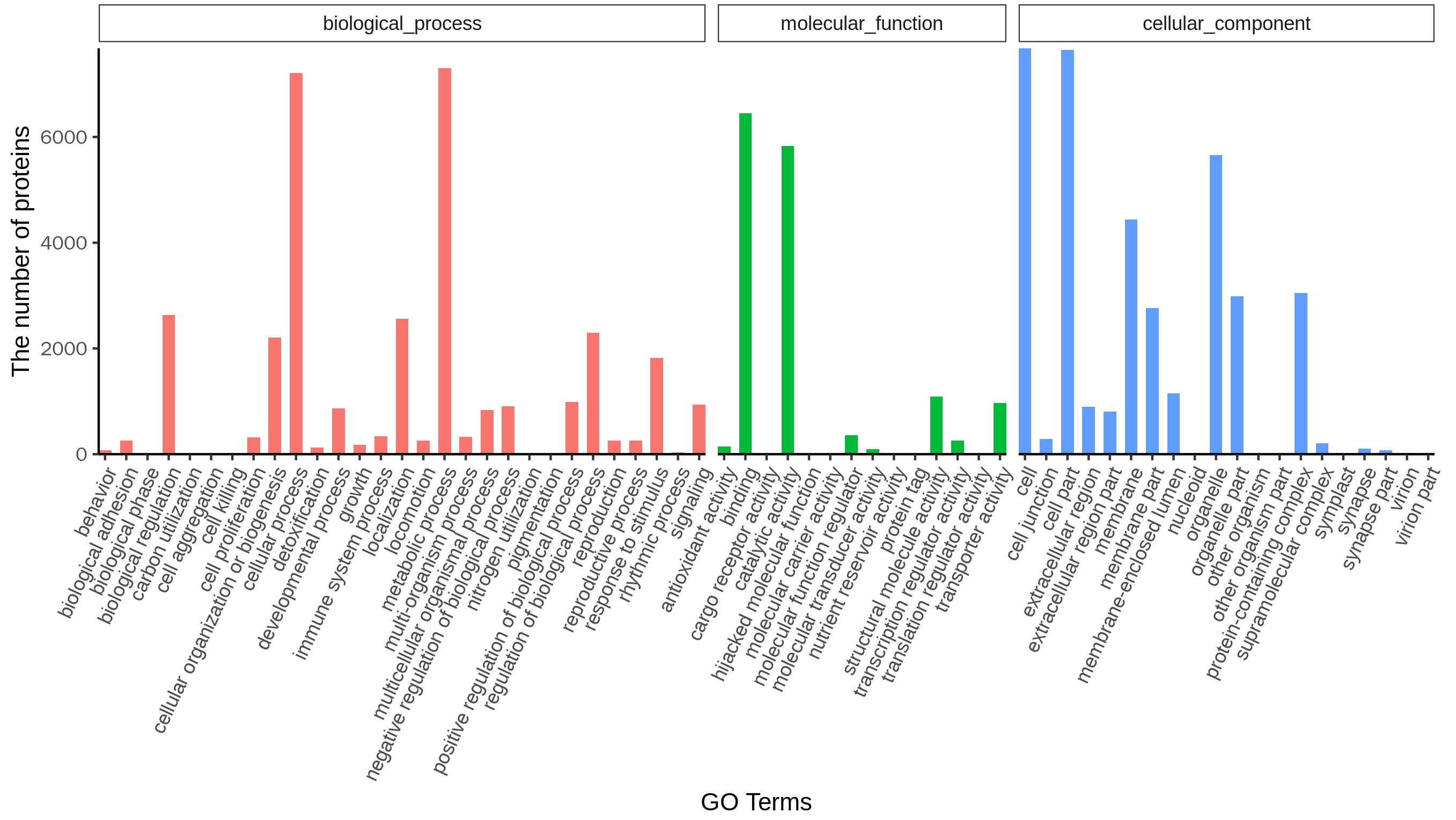

Supplement: Supplementary file 1 [file life-12-01337-s001.zip › Figure S1. Gene ontology classification of unigenes.jpg]

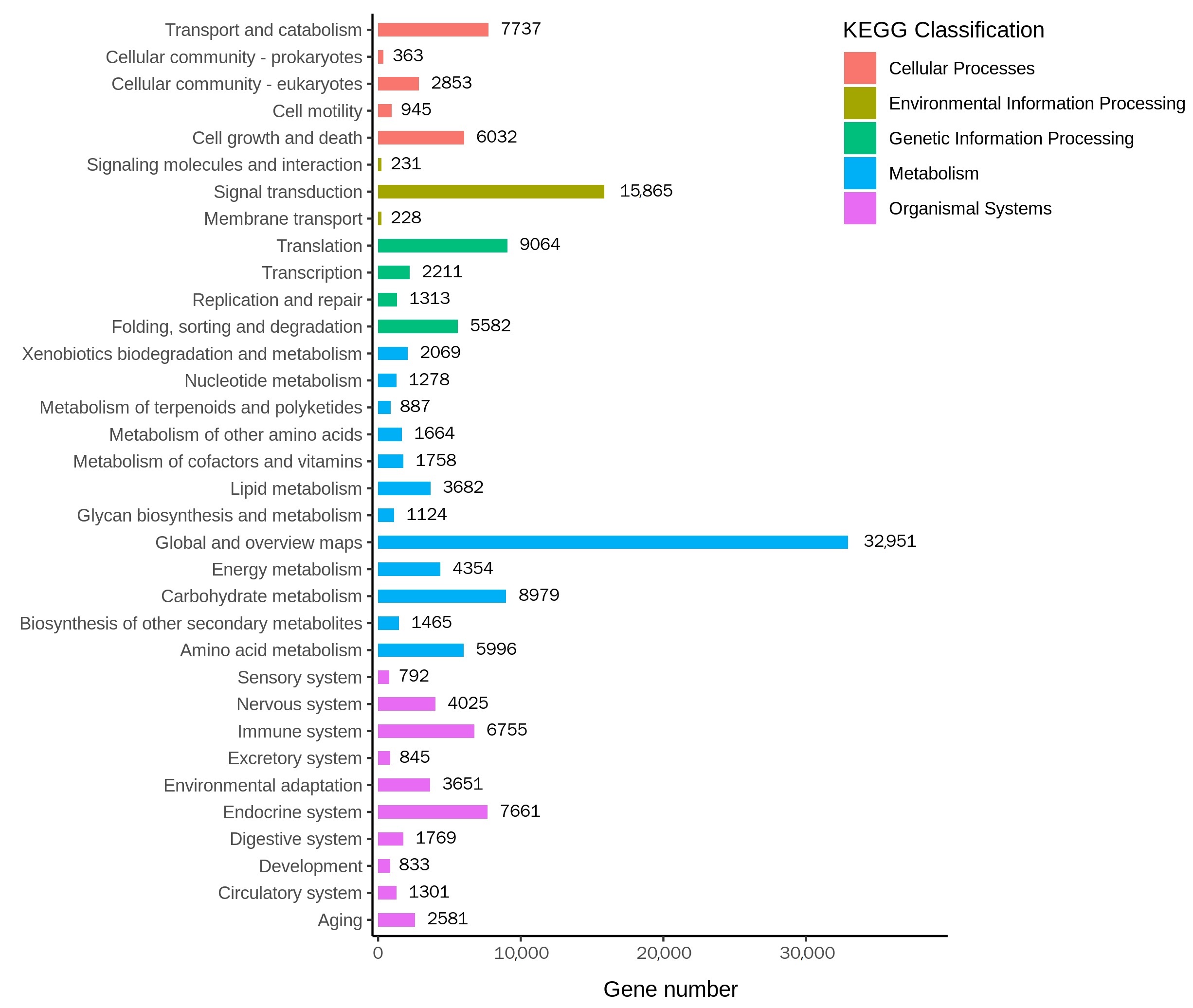

Supplement: Supplementary file 1 [file life-12-01337-s001.zip › Figure S2. KEGG classification of unigenes.jpg]
